# Visual authoring of virtual reality conversational scenarios for e-learning

**DOI:** 10.1007/s10209-022-00934-3

**Published:** 2022-10-20

**Authors:** Rubén Baena-Perez, Iván Ruiz-Rube, José Miguel Mota, Anke Berns, Antonio Balderas

**Affiliations:** 1 Computer Science Department, Universidad de Cádiz, Av. de la Universidad de Cádiz, 10, 11519 Puerto Real, Spain; 2 Department of French and English Philology, Universidad de Cádiz, Av. Gomez Ulla s/n, 11003 Cádiz, Spain

**Keywords:** Chatbots, End-user development, E-learning, Learning analytics, Virtual reality, App inventor

## Abstract

The COVID-19 pandemic has led to face-to-face activities being developed in a virtual format that often offers a poor experience in areas such as education. Virtual Learning Environments have improved in recent years thanks to new technologies such as Virtual Reality or Chatbots. However, creating Virtual Learning Environments requires advanced programming knowledge, so this work is aimed to enable teachers to create these new environments easily. This work presents a set of extensions for App Inventor that facilitate the authoring of mobile learning apps that use Chatbots in a Virtual Reality environment, while simultaneously monitoring of student activity. This proposal is based on integrating block-based languages and Business Process Model and Notation diagrams. The developed extensions were successfully implemented in an educational app called *Let’s date!*. A quantitative analysis of the use of these extensions in App Inventor was also carried out, resulting in a significant reduction in the number of blocks required. The proposed contribution has demonstrated its validity in creating virtual learning environments through visual programming and modelling, reducing development complexity.

## Introduction

The COVID-19 pandemic situation has led to increased interest in applying emerging technologies in the field of education. According to Google Trends, search peaks about e-learning coincide with the pandemic’s beginning in April 2020.^1^ Given the restrictions on attending classes and experiencing learning activities outside, interactive technologies, such as Conversational Agents (CAs) and Virtual Reality (VR), have brought special interest to the educational community.

Nowadays, e-learning is widely adopted in the education field, using Virtual Learning Environments (VLEs). These environments combine several tools that allow learners to access, interact, and be evaluated on the content offered and which have been demonstrated to increase their knowledge, confidence, and satisfaction, the latter being important for learner engagement and knowledge acquisition, affecting their information retention capacity [1].

The use of these VLEs provides a large amount of data, offering a vision of the learning process. The areas of Educational Data Mining (EDM) and Learning Analytics (LA) are widespread [2, 3], and aim to understand learners and improve the quality and efficiency of learning processes by analysing the data collected in the learning environment [4]. Although these data are currently available, it is difficult for teachers and school administrators to interpret them, since they do not know the techniques needed to extract and process them [5].

A relevant technology for providing interactive learning scenarios is VR. This uses computer graphics systems in combination with various display and interface devices to provide the effect of immersion in an interactive 3D environment [6]. Developing solutions based on artificial 3D scenarios makes software development more complex. Furthermore, there is a lower perception of reality than when using real images [7]. For that reason, 360$^\circ$-based environments have been used in the current study.

Chatbots and CAs use computational linguistics techniques to interpret and respond to statements made by users in ordinary natural language. These systems, with voice or text chat functions, have been improved by advances in Automatic Speech Recognition (ASR) and Natural Language Processing (NLP), thanks to which it is possible to obtain almost instantaneous responses with a high degree of naturalness [8]. The potential of Chatbots for mentoring students has been researched, with the conclusion that their current development does not have a clear pedagogical focus on improving and supporting learning [9]. One emerging field of Chatbot applications are as Pedagogic Conversational Agents (PCAs), which are autonomous characters that cohabit with students in a learning environment and which are usually employed to create rich learning interactions [10].

Every new interaction style requires the involvement of various expert profiles, including programmers, developers, and human-computer interaction experts. That restricts the creation of interactive learning materials and activities to non-experts, not to mention the worldwide scarcity of computing professionals and software developers. To alleviate such deficiencies, Visual Programming Languages (VPLs) have been researched in the End-User Development (EUD) field to allow non-expert users to develop their own software solutions with no need of having specialized programming skills [11]. VPLs mostly use block-based and flowchart graphical representations to allow non-expert users to program without having to learn the syntax of a traditional programming language.

Therefore, in this work, the following hypothesis is raised: flow chart diagrams and block-based languages can be used in combination with CAs to easily develop learning environments based on multimodal VR scenarios. These scenarios support LA, enabling educators to track learners’ activity.

A set of extensions developed for the MIT App Inventor (AI2) mobile application authoring platform [12] is presented as a contribution to this work. Such extensions enable the authoring of a VR environment to interact via a chatbot, recording the app user’s activity and defining the app’s behaviour by employing a Business Process Model and Notation (BPMN) diagram. The extensions were developed to facilitate the use of the app with a type of VR glasses that requires a smartphone. Nonetheless, the extensions allow using the app without any need to employ VR glasses.

The rest of the document is structured as follows. Section 2 presents the research background and related works. Section 3 describes the contribution made, consisting of a set of extensions to the AI2 development environment. Section 4 shows an illustrative example of how to design a virtual scenario with the provided tools and provides a quantitative assessment of the required building blocks when creating a demo application with and without the new AI2 extensions. Section 5 includes a case study with a mobile app to enable foreign language students to immerse themselves in real-world conversations with native speakers of the target language. The final section discusses the conclusions and future work.

## Background and related works

This research aims to demonstrate that it is possible to easily create multimodal VLEs that integrate LA using flowcharts and visual block-based languages. The following is a description of the concept of VPL and its different types, as well as of the latest advances experienced by VLEs.

### Visual programming languages

The development of software solutions involves a considerable effort for those users who are not experts, limiting access to the use of the latest technological innovations. To solve this problem, in recent years, EUD has recently experienced a renewed interest, providing the necessary tools for non-expert users to develop their software solutions through adapted languages [11].

Programming with VPLs does not require knowledge of textual programming syntax and provides a more visually stimulating environment, having a direct impact on user motivation [13]. Such languages are commonly used in educational settings for promoting students’ development of computational thinking [14]. The suitability of block-based environments for novice programmers has already been demonstrated in different studies. Broll et al. [15] propose a web development environment based on Snap! that adds distributed programming capabilities. Kyfonidis et al. [16] present a block-based programming environment focused on learning the C language. Finally, Rao et al. [17] propose a visual programming environment for education in data science.

VPLs can be classified into two categories: imperative languages and flow-based languages [18]. With the imperative languages, namely, block languages, the user creates their software by assembling different puzzle pieces intuitively. Within this category, Scratch, Snap! and Google Blockly stand out. Scratch enables novice programmers to create apps through its block-based language. However, the purpose with which these languages were created entails some limitations when it comes to use the latest technological innovations as, for instance, VR. On the other hand, with the flow-based languages, the models are created by joining different nodes and graphic lines representing the data flow or the transition between different states. Within this category, languages such as BPMN, PetriNet, DRAKON or UML activity diagrams can be found, among others [19].

### Virtual learning environments

Recent technological advances, such as the miniaturization of hardware or the significant increase in the information provided by electronic devices, have led to the emergence of new systems to improve users’ experience of their environments. These systems offer support for different interaction modalities and proactively help users [20].

In the educational field, the appearance of these systems has allowed the introduction of more attractive, interactive, and customized formats, moving from the traditional web environment to multimodal VLEs. In addition, these VLEs provide a large amount of data that can be extracted and used thanks to the areas of EDM and LA, providing information on both the quality of the learning itself and the environment in which it takes place.

Among the multimodal interactions offered by this new generation of VLEs, those that make use of VR stand out, thanks to their ability to enhance or complement traditional analog learning spaces. VR offers the user an illusion of reality that allows them to immerse themselves and interact as if they were there. According to the value-control theory of achievement emotions [21], this immersion increases positive values in task and object learning.

The applications of VR in education are largely biased towards simulation and training [22], with effective results in improving gains in learning outcome [23]. In this sense, previous studies that validate the benefits of using VR in educational environments have been found, such as the one conducted by Merchant et al. [24]. This study concludes that VR learning reflects higher student performance compared to traditional learning.

Interface devices such as head, eye, and other body parts recognizing controllers and trackers are often used to interact within VR scenarios. Users’ operation with these devices must be trained beforehand, which increases the complexity of the interaction process. Using device-based interactive solutions in VR poses human memorization and training issues, as well as sensory problems, since the number of functions provided by a VR scene increases. To overcome this problem, learning designers can implement multimodal interactions based on voice control, possibly combined with other interaction styles [25].

Another of these types of multimodal interactions are Chatbots. The use of Chatbots has experienced a boom in recent times, thanks to important advances in the fields of NLP and ASR [8]. These advances make it possible to obtain an almost instantaneous response to a question with a high degree of naturalness. As a result, ASR-powered voice interfaces that use Chatbots are preferred by users, even if they are more time-consuming than other traditional means of data input [26]. Moreover, in certain situations, they can outperform a traditional keyboard and mouse interface in terms of speed [26].

Regarding the authoring of these VLEs, there is a growing trend in the literature to adopt a human-centred approach, where the interaction opportunities, functions, and attributes of the system are defined by the intended users themselves [27]. Among these works is the one presented by Saunier et al. [28], in which the authors propose a methodology for designing VR environments using UML. There are other proposals, such as the one made by Wolfartsberger et al. [29] or by Mota et al. [30]. While the first one suggests a solution based on Unity3D in which VLEs can be generated under the action authoring approach, the second one illustrates how the use of the Visual Environment for Designing Interactive Learning Scenarios (VEDILS) tool, a fork of AI2, enables users to create VLEs that make use of Augmented Reality (AR) using its block programming language. Concerning the works covering the authoring of VLEs based on Chatbots, the literature review revealed one study, by Schmulian et al. [31], in which a teacher-oriented visual development environment is proposed to create Chatbots.

The emergence of these new systems implies creating or adapting LA tools that make it possible to analyse all this large amount of data derived from these new event types. In this vein, Santamaría-Bonfil et al. [32] propose the creation of a student model in VR environments using a series of classifiers and three minimum evaluations. Fernández-Gallego et al. [33] also present a framework oriented to 3D educational virtual worlds, where through OPENET4VE, the events of such a platform are recorded and processed, allowing teachers to know and make changes in the learning workflow. Although there are proposals for the use of learning analytics in VR environments, no previous work has been found in the literature on VR environments that addresses chatbots and learning analytics.

Despite having found proposals to create multimodal VLEs that make use of VR or Chatbots, such tools are limited or not aimed at teachers; hence delaying the adoption of this new type of VLEs. In addition, these environments generate new types of events for which proposals have been found to exploit this data. However, none of these proposals follows a human-centred approach.

## Authoring of interactive scenarios with visual modelling and programming

The objective of the current study is to materialize how flow chart diagrams and block-based languages can be combined with NLP platforms to develop conversational learning experiences. In this section, we first explain the tools used for visual programming and workflow modelling. Then, we describe the execution components created for the programming tool.

### Authoring tools

The visual programming tool and the BPMN modelling environment for defining the app behaviour are now detailed.

#### App visual programming

AI2 is an open-source platform that enables users to develop Android mobile applications by using the Google Blockly language and following a human-centred approach. The AI2 architecture is composed of several modules. For the user interface design, AI2 provides a Google Web Toolkit (GWT) application and a Blockly editor for programming the behavioural logic of the apps. There is also a compiler server to convert the existing design and logic into an exportable Application Package (APK) or Android App Bundle (AAB) file. In addition, AI2 has an interpreter for debugging apps and a module with all components available for end-users to develop applications. VEDILS is the fork of AI2 that includes some additional components for creating augmented reality (AR) apps in combination with other Features [30]. To support this research and to develop VR-based mobile learning environments with voice interaction, several software extensions were created for VEDILS. These extensions serve workflow running, VR rendering, voice dialogue managing, and user experience tracking.

#### Workflow modelling

BPMN processes can be deployed and enacted through specific execution platforms that can automate and coordinate task execution. The sequence of mobile VR scenes is modelled with BPMN so that its execution can be automated. To do that, specific semantics have to be defined for the core elements of the BPMN standard that have been used, namely tasks, which represent atomic activities within a process flow. For our purposes, the following meanings are considered for some task specializations:

- Service Task: delivers a 360$^\circ$ video to immerse users in realistic contexts;
- User Task: launches the acquisition of a voice message from the user and its understanding via an agent from an NLP platform;
- Manual Task: with this task, the app will ask for a confirmation action from the user via a button in a non-VR dialog interface.

Through the *documentation* BPMN property, service tasks are linked to the corresponding 360$^\circ$ videos, manual tasks are set with the messages to display to the user, and user tasks are bound with the variables which store the result from the voice agent. Any BPMN-compatible tool can be used to model the learning scenarios, but the current implementation has been tested with the client-side web application provided by the BPMN.io project, which is built and maintained by Camunda [34]. If a more usable workflow modelling component prototype were required, a software adaptation of Camunda interface having purpose-specific tasks would be needed. For the sake of simplicity and without loss of generality, we have used the Camunda tool employing standard tasks that have been adapted with the operational semantics explained above.

### Execution components

The created components, which are now detailed, enrich the visual language with new blocks that add functionalities for workflow execution, VR/360$^\circ$ rendering, voice dialog management, and user experience tracking.

#### Workflow execution component

**Fig. 1:**
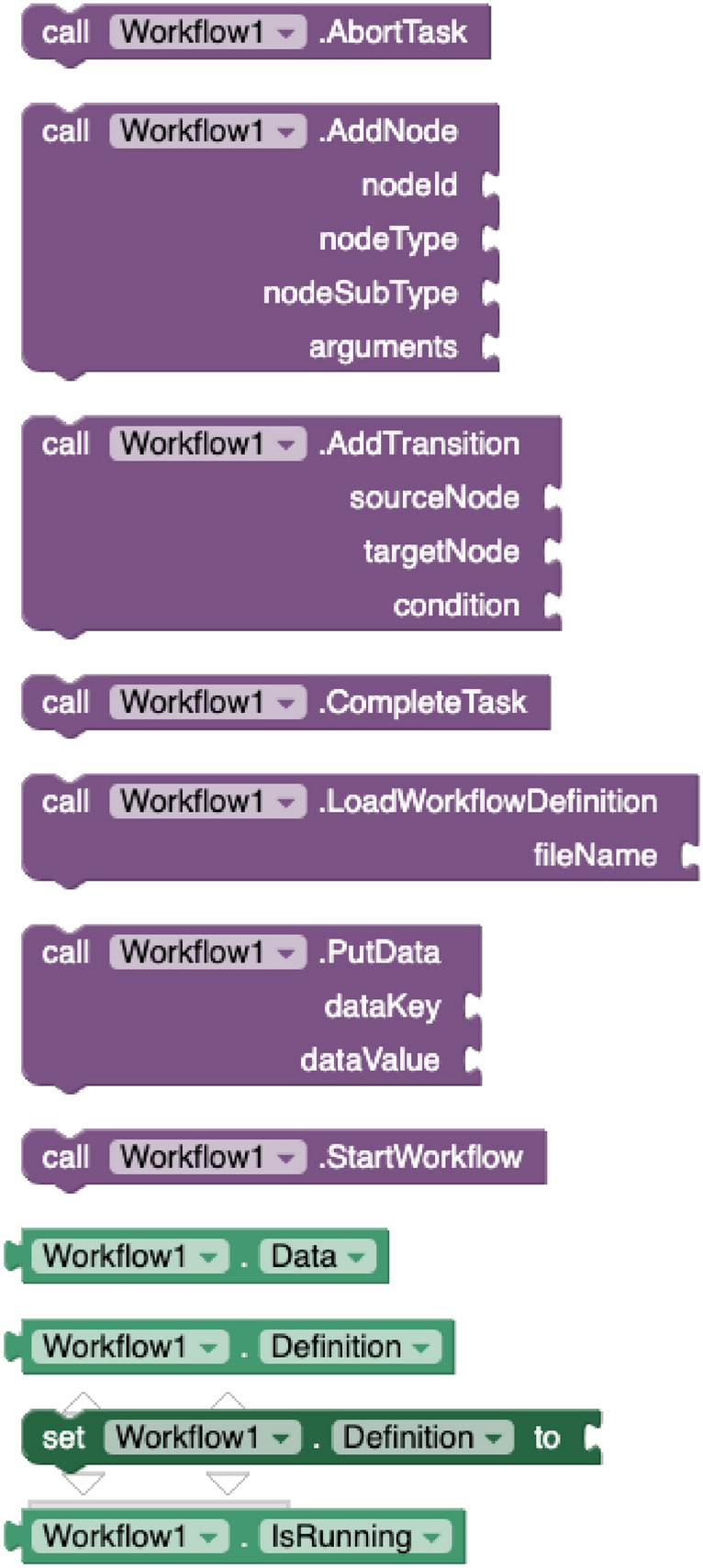
Workflow methods and properties

**Fig. 2:**
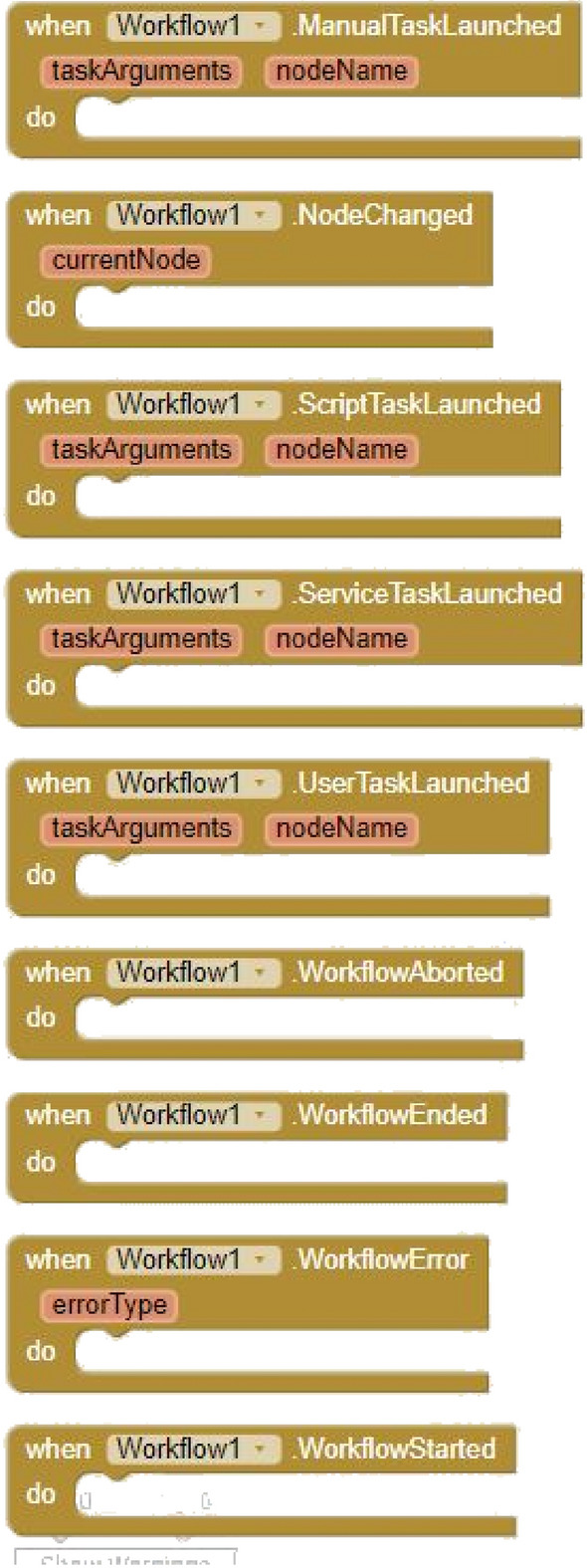
Workflow events

This component enables mobile apps to orchestrate the set of actions to perform according to the user’s responses. This component provides a property to select the XML file containing the *definition* of the BPMN model. Regarding its methods, it provides blocks to *start* and *abort* the process, go forward in the flow by *completing* the current task, *put data* into a transient user’s hash map (accessible from the *Data* property) and *add nodes* and *transitions* to the model dynamically. Figure 1 shows the corresponding blocks. The component has several event handlers (see Fig. 2) to manage when the flow has *started*, successfully *ended*, programmatically *aborted* or with *error*. The events *UserTaskLaunched, ServiceTaskLaunched, ScriptTaskLaunched* and *ManualTaskLaunched* are fired every time the current execution reaches the corresponding task type, whereas the event *NodeChanged* was developed to debug the workflow execution. Under the hood, the component has been developed in pure Java and utilizes the BeanShell and XmlPullParser libraries.

#### VR/360$^\circ$ rendering component

**Fig. 3:**
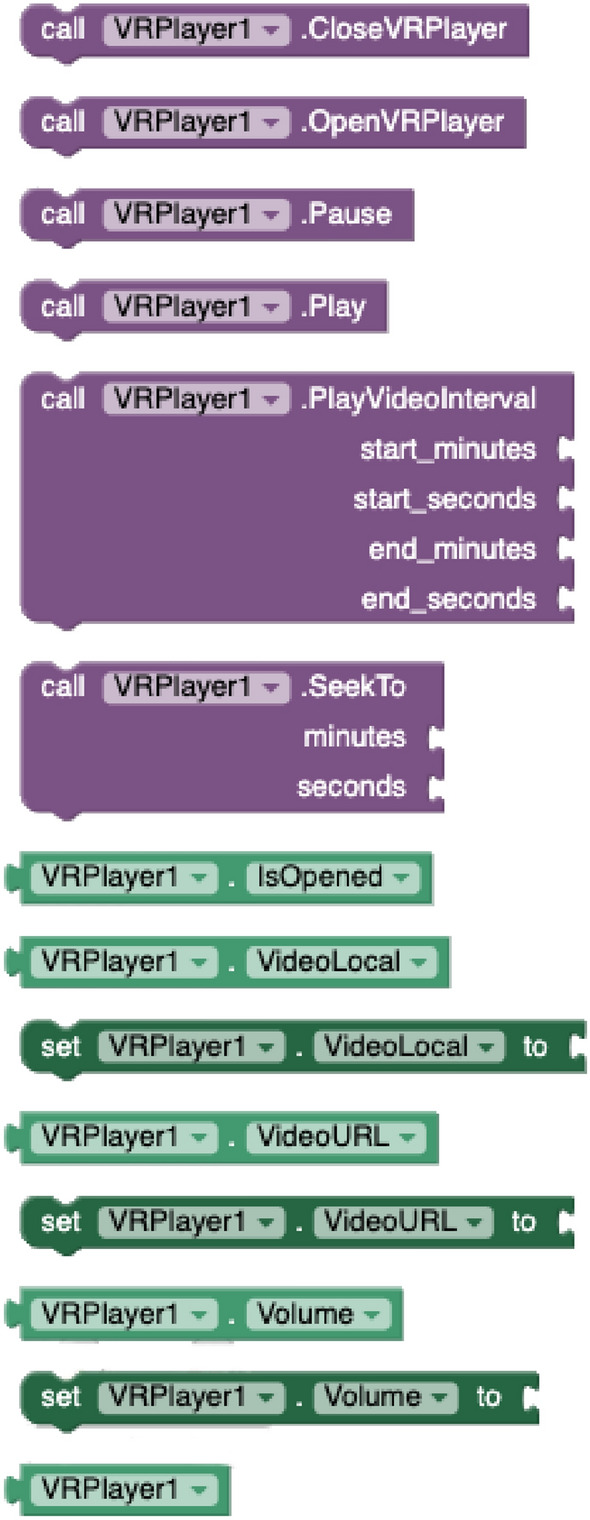
VR methods and properties

**Fig. 4:**
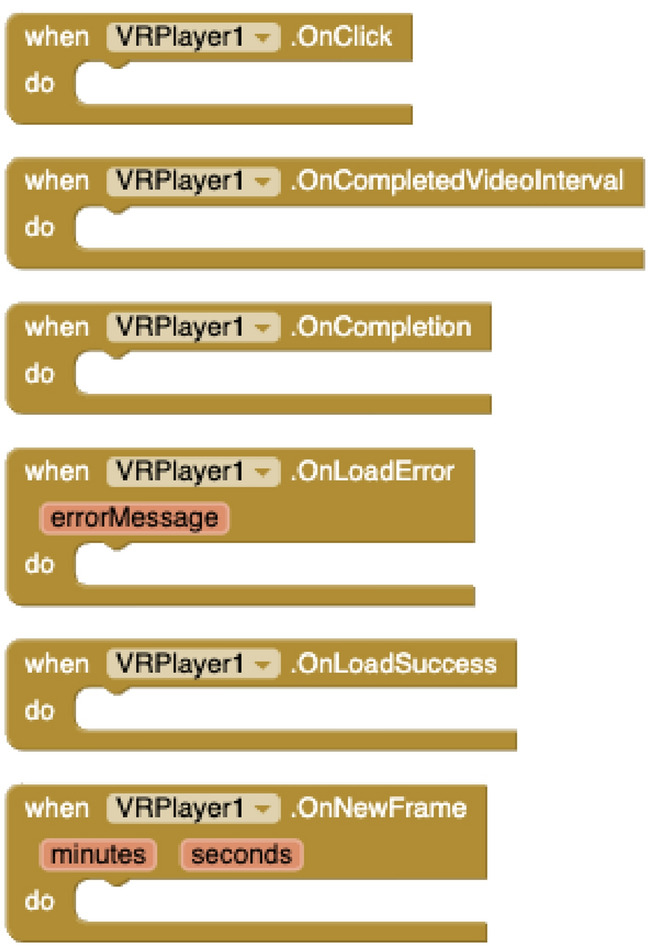
VR events

This is an extension to support stereo delivery of VR panoramic videos (VR/360$^\circ$) hosted on video streaming platforms from mobile applications. To allow that process to happen, developers have to set the video streaming *identifier* (YouTube videos are currently supported) and the audio volume. In runtime, apps can *open* and *close* the 3D spherical player, *play* and *pause* the delivery and *seek to* or *play video interval* a given timestamp. The player also notifies through events for every *new frame*, at the end of the video, and when the user taps (*onClick*) on the screen. All of these blocks are shown in Figs. 3 and 4. The 360$^\circ$ VR rendering component has been developed using the Google VR SDK.^2^

#### Dialog component

An NLP platform is required to process voice interactions. Although many platforms allow creating conversational agents, the current implementation has been tested with Google’s DialogFlow cloud-based NLP platform. A set of intents and entities must be defined for the virtual scenario.

**Fig. 5:**
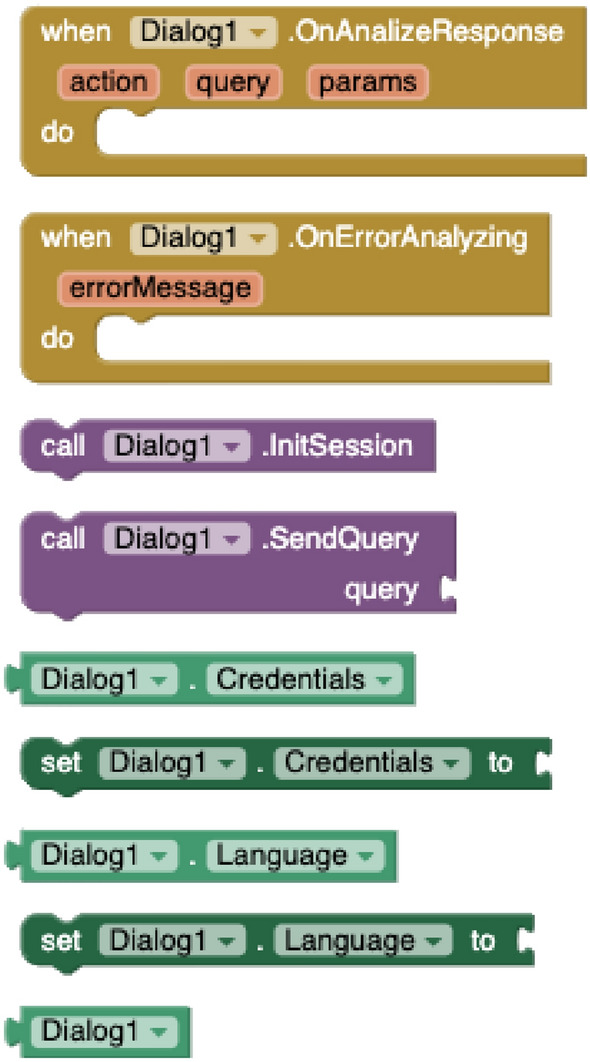
Dialog blocks

With the *Dialog* component (see Fig. 5), apps created with VEDILS can listen to the users’ voice inputs and analyse them according to certain predefined rules in a conversational agent running on the NLP platform. For the DialogFlow implementation, developers have to select first a JSON file with the developer’s *credentials* in the Google Cloud infrastructure and indicate the base *language* of the conversational agent. The component provides methods to *init a session*, *start* and *stop* voice listening, analyse the transcription of the voice (*SendQuery*), and *get a parameter from the response*. The mobile app is notified by receiving three different events from the conversational agent: when *finishing* listening, when a *response* is obtained, and when an *error* is produced.

#### User experience tracking components

To capture data about student learning experiences, the *Experience Tracker* extension was developed. This component creates and emits xAPI statements to any xAPI-compliant system. The component supports the following properties: *RealTime* to specify whether data must be immediately sent when the xAPI statement is prepared; *Batchtime*, to specify the time interval to submit statements when the above property is disabled; *OnlyWifi*, to indicate whether data transmission must be performed via Wi-Fi or with any data connection type; *RecordStoreURL*, to define the Learning Record Store (LRS) connection endpoint; and *RecordStoreToken*, to set the corresponding access token.

**Fig. 6:**
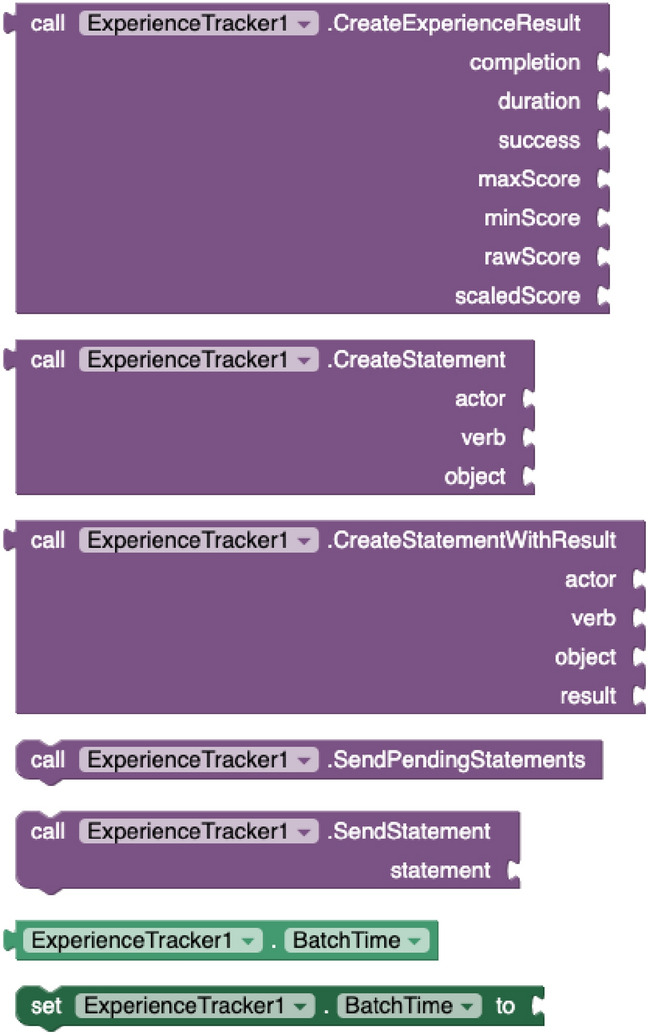
Experience Tracker blocks

**Fig. 7:**
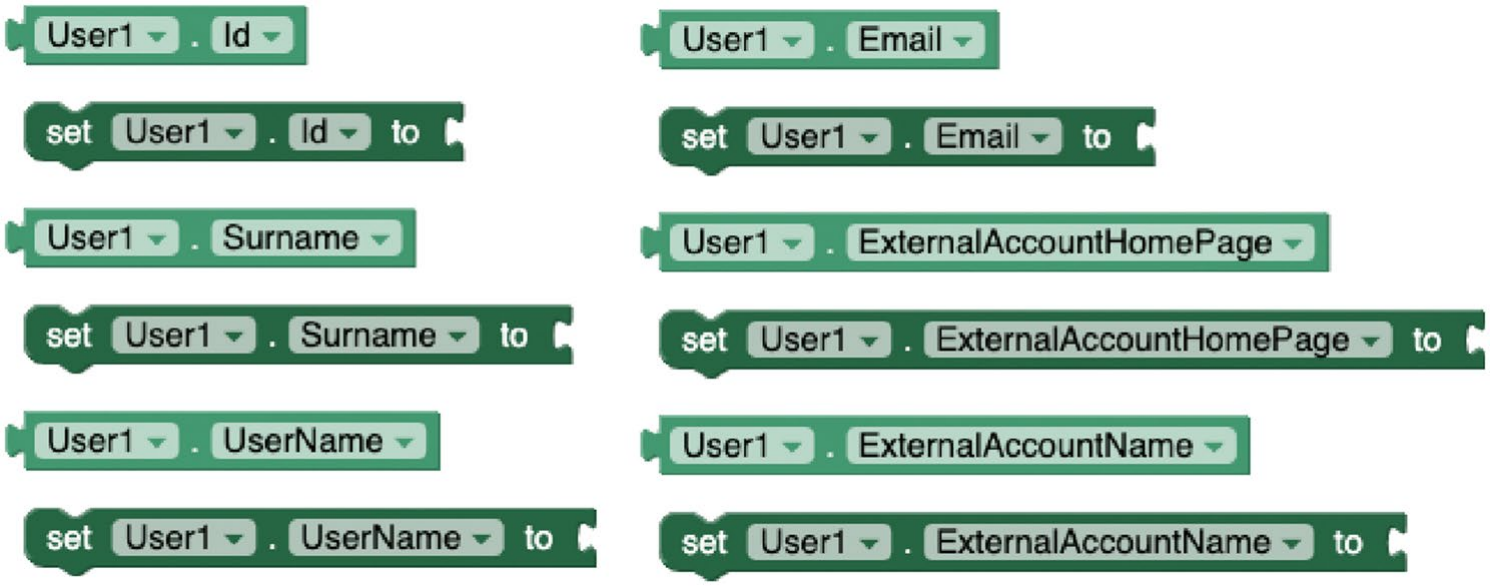
User blocks

The component provides blocks to *create statements* conforming to the xAPI scheme, through the actor, verb, object, and (optionally) result parameters. The sentences are issued to the LRS by using the *send statement* and *send pending statements* methods (see Fig. 6). The actor parameter represents the learner who is experiencing learning, and they are defined through another component, called *User*. This new component (see Fig. 7) has properties to set a user *identifier* and *name*, their *email address*, as well the properties *ExternalAccountHomePage* and *ExternalAccountName* to identify the user on other platforms, such as Twitter. The verb parameter must be a URI of an xAPI verb, such as ”http://adlnet.gov/expapi/verbs/experienced”. The object parameter can be supplied with a URI of a specific activity, a *User* corresponding to another actor or another previously created statement in the case of voiding. Finally, a fourth parameter (result) can be included when creating statements to provide the outcome of the learning activity. For that, the component has an additional method to *create experience result* by providing the completion, duration, success, maxScore, minScore, rawScore, and scaledScore values.

## Evaluation

This section presents an example of a simple VEDILS application using the components described above to verify the feasibility of the contribution. Afterwards, a quantitative analysis to check the ease of use of the provided components is described.

### An illustrative example

**Fig. 8:**
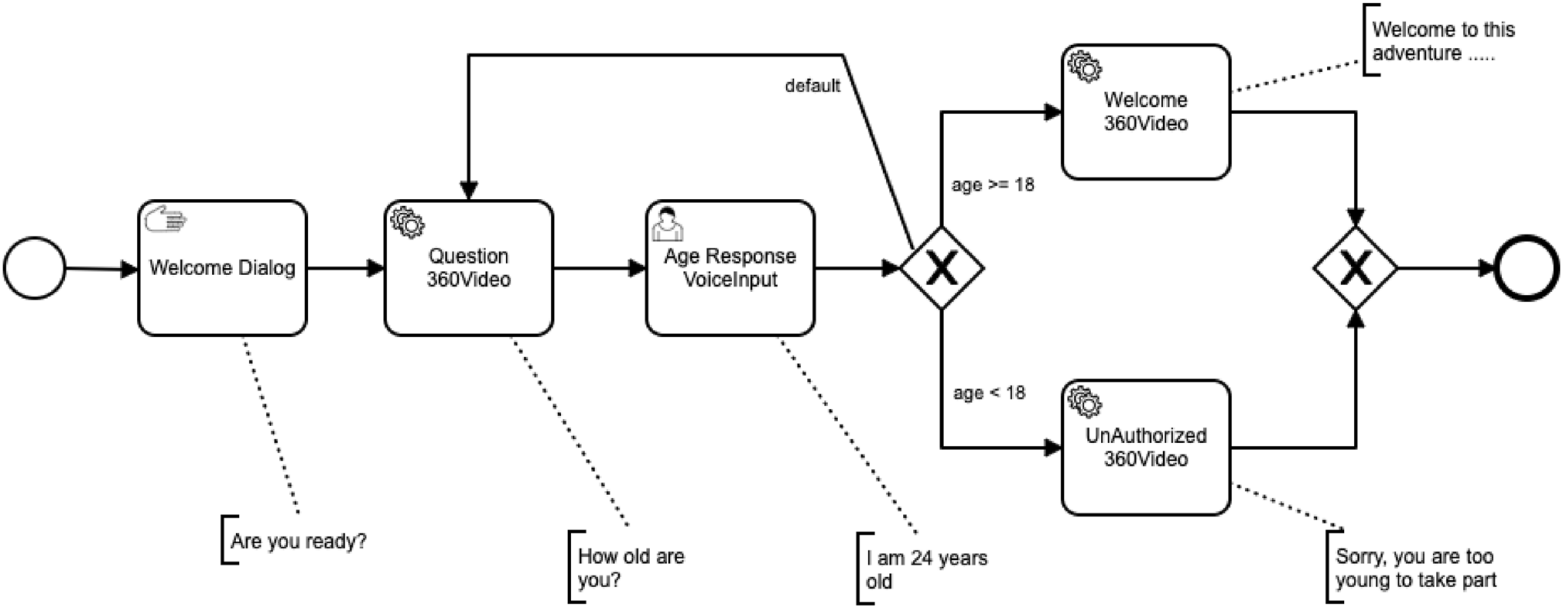
Example of multimodal virtual environment

The following example depicts how the new execution components can be put together to create, run and track an integrated VR-based conversational experience for e-learning. Figure 8 depicts the learning scenario. With this model, the environment firstly launches a 2D modal window for giving information to the user and asks for confirmation to start. Then, the user is immersed in a real situation by watching a panoramic video with VR headsets. During the delivery of the video, the user is asked to respond to a simple question about his/her age. Then, the device enters on listening mode and processes the user’s voice input and, depending on the response (higher or lower than 18 years), the user will see a different video based on a new scenario. In case the user has provided an unexpected answer, the app will immediately return to the previously delivered question video and ask the user to revise his/her answer

**Fig. 9:**
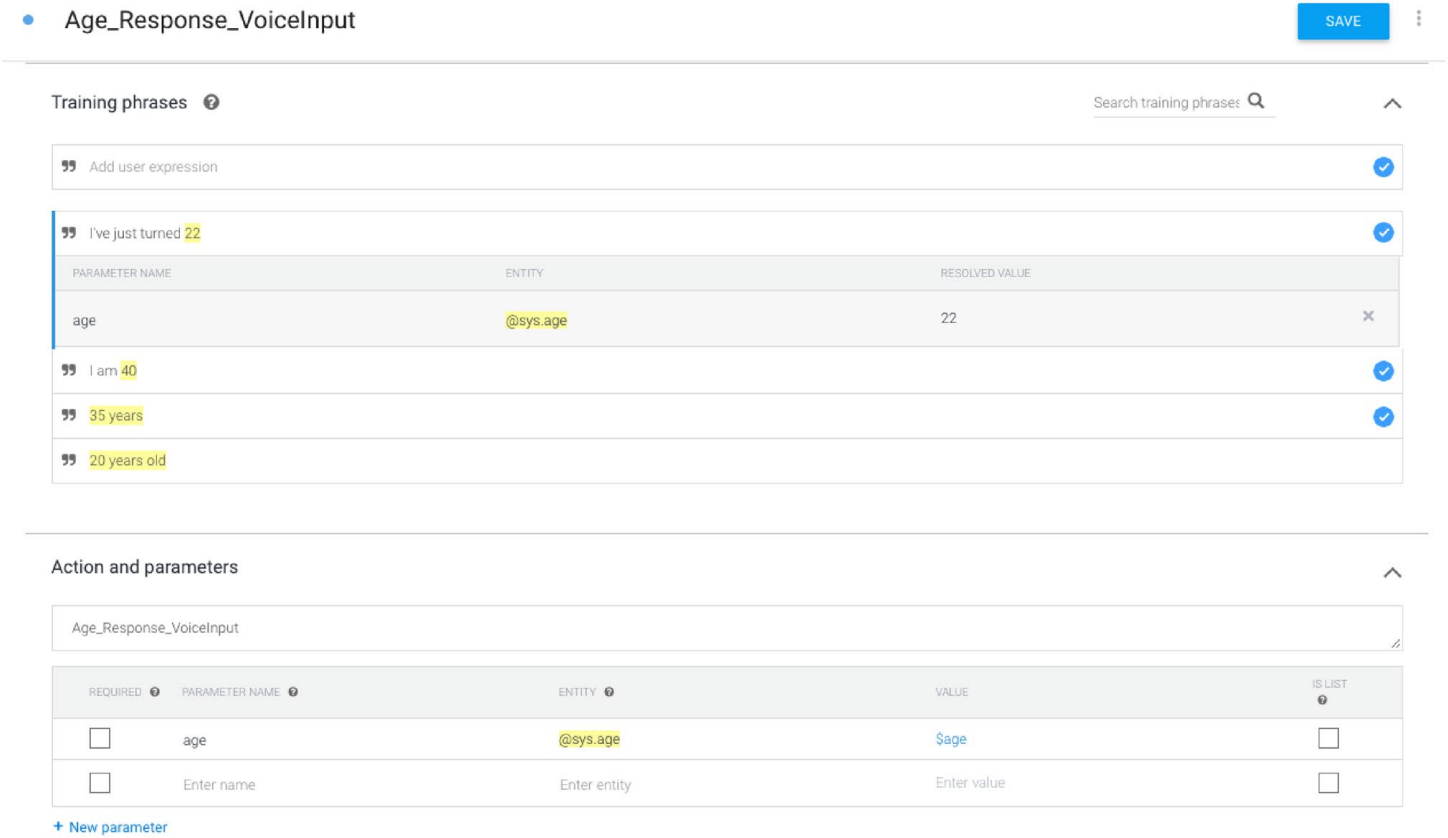
DialogFlow web interface with default intents

A DialogFlow agent for the VR scenario from Fig. 8 is shown in Fig. 9. This agent consists of two intents, one for managing situations where the agent does not recognize an end-user expression and another one for managing responses for the age-related question. The app-specific intent is pre-populated with training phrases such as ’20 years old’, ’35 years’, or ’I am 40’. Developers can manually customize the set of training phrases for each intent.

**Fig. 10:**
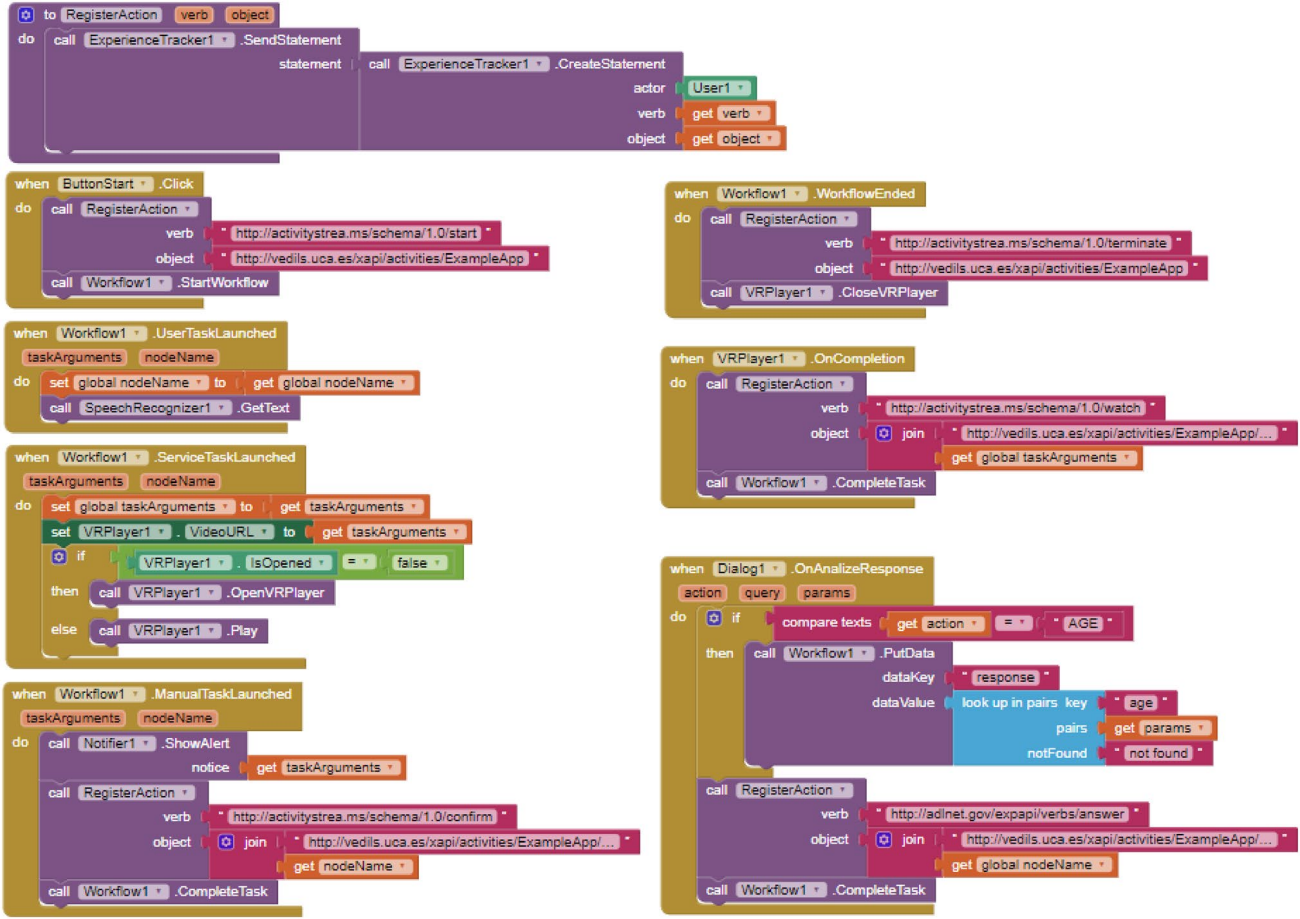
Example blocks for managing the workflow and the VR features

The building blocks to address all these aspects are presented in Fig. 10. This example aims to build a VR mobile application that supports the environment modelled in Fig. 8 and reports on the actions performed by the user. The virtual environment is started by touching a button on the screen. That event launches the BPMN engine, reports the start of the application, and opens the VR player that switches the application screen to stereo mode. Notifications about the performed actions are carried out by the procedure *RegisterAction*. The given semantics for the input model is implemented in the event handlers *UserTaskLaunched, ServiceTaskLaunched* and *ManualTaskLaunched*. When a manual task is reached, the app displays an alert window to display a message and wait for the OK button to be pressed. Then, the current task is completed, the action performed is reported, and the workflow moves forward. When a service task is reached, the app reads the URI of a 360$^\circ$ video of the BPMN model and plays it. Once the video has finished, the action performed has been reported and the respective task was completed, the workflow can progress. When a user task is reached, the application starts listening to the user’s voice. As the output is parsed, data is extracted and stored in the user’s hash map, the action performed is reported, and the current task is completed to move the workflow forward. This scenario takes alternative paths based on this data. Upon reaching the final event of the BPMN model, the player is closed, the completion of the application is reported, and the user’s age value is captured during execution.

### Quantitative evaluation

To conduct the study to check the suitability of the provided components, we implement a quantitative evaluation. Two previous implementations of the app were carried out in two different development environments to perform this analysis. For the first implementation, we used VEDILS, that is, an instance of AI2 with the new execution components, while, for the second implementation, we used a bare instance of AI2 with none of those components.

Briefly, the main features provided by the app are the following: (i) loading and playback of different VR scenarios; (ii) voice interaction with the NLP DialogFlow platform; (iii) recording of the actions performed by the user in the app; and (iv), determination of the behaviour of the app based on the BPMN diagram. Once the two versions of the app were developed, we count the number of blocks required to develop those features (see Table 1).

**Fig. 11:**
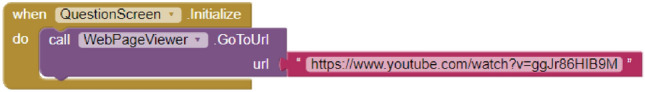
Blocks used to reproduce a VR scene without specific VR components

Regarding the first feature, by default, AI2 does not have components to reproduce a VR environment, so our proposal fills this need. The only way to do that to some extent is by launching an external video player. However, the app would lose control of the video playback and, hence, environment realism. Figure 11 shows that approach using the *WebViewer* component included in AI2, renamed *WebPageViewer*.

**Table 1:** Number (n) of blocks required to replicate the illustrative app with the bare AI2 and with AI2 equipped with the provided components

Quantitative evaluation		
Feature	AI2 (n)	AI2 + new components (n)
Virtual Reality	﻿4	8
Voice interaction	53	10
User activity tracking	132	31
Scene flow management	43	28
Total	232	77

**Fig. 12:**
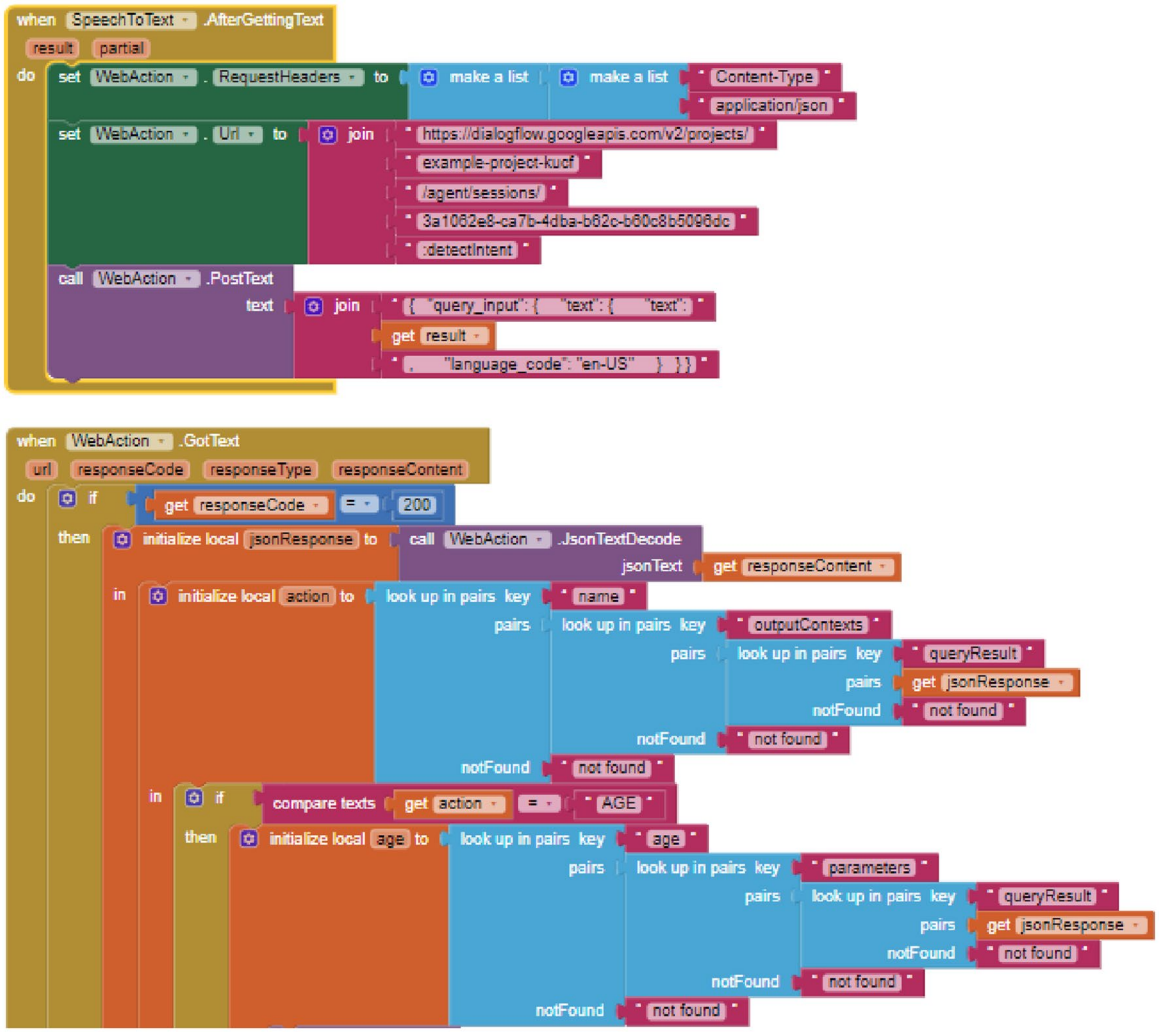
Blocks used to communicate with DialogFlow REST API using AI2 without VR components

For the second feature related to the voice interaction, with the bare AI2, 53 blocks were used. Such an overload of blocks is necessary to make an HTTP POST request to communicate with the DialogFlow REST API and process the response in JSON format. Figure 12 shows that the *Web* component included in AI2 (renamed *WebAction*) was used for HTTP requests. For sending the POST request, the *PostText* block was used, to which the text of the request has to be issued to the DialogFlow REST API. For processing the server results, the *GotText* event block was used. In addition, a conditional block is used to check that the request has been fulfilled correctly. If so, the response content is transcribed into JSON format using the *JsonTextDecode* block, which belongs to the *WebAction* component. In addition, for each of the response parameters, 3 *look up in pairs* blocks have to be used to obtain the user responses, which means a total of 10 blocks for each parameter. With the new *Dialog* component, only 10 blocks were required, that is 430% fewer blocks.

**Fig. 13:**
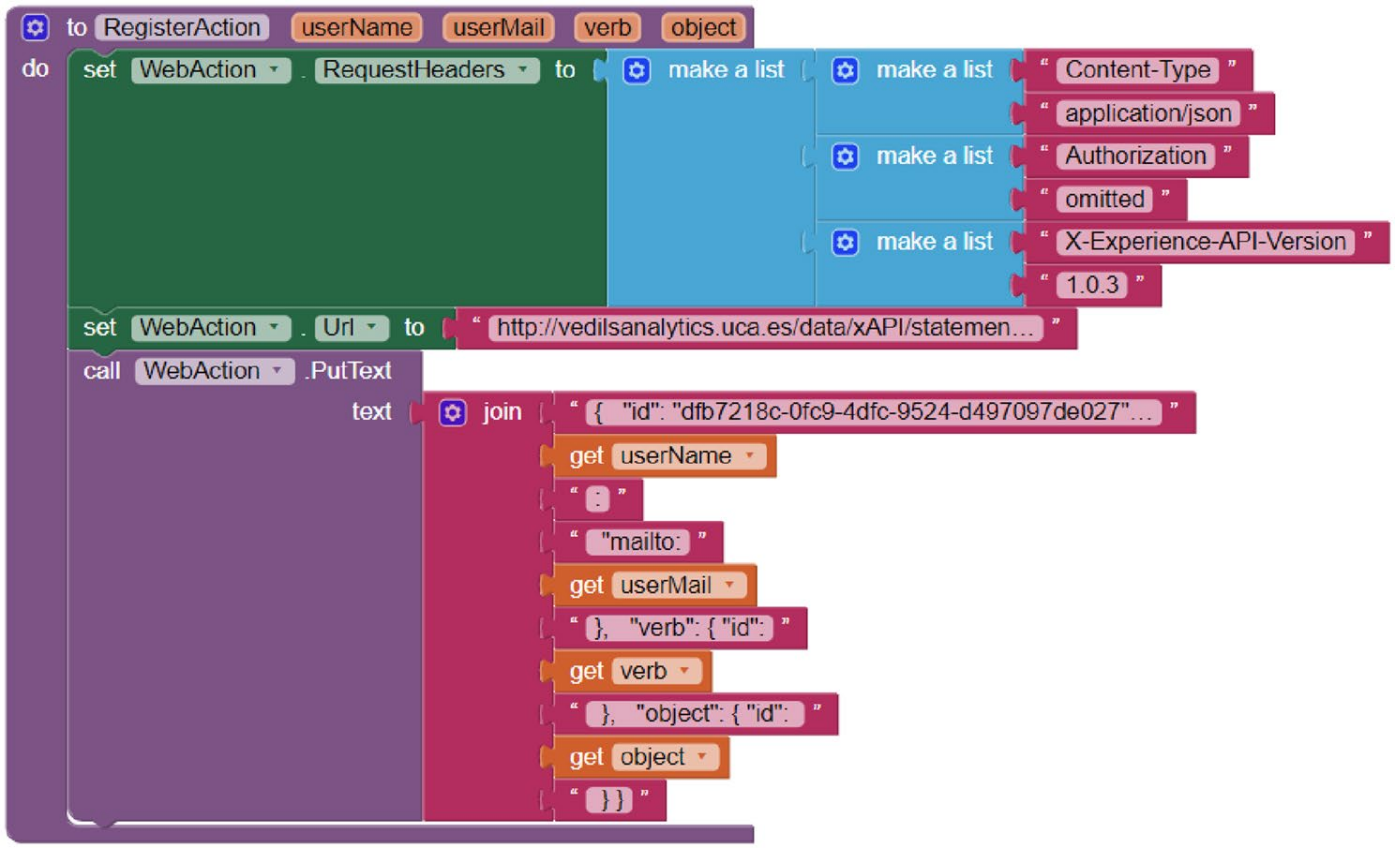
Blocks used to record user actions using AI2 without the new components

For supporting the third feature, related to the user tracking with the xAPI specification, with the app developed solely with the bare AI2, it was necessary to create a procedure to execute a PUT HTTP request to track each action. Figure 13 shows how this procedure, called *RegisterAction*, has a series of input parameters to issue the correct xAPI statement. Within this procedure and in order to perform the HTTP request, the block *PutText* belonging to the *Web* component (renamed as *WebAction*) is used. Furthermore, this procedure has to be repeated in each *Screen* when data tracking is required. 132 blocks were used in the application that was developed by using solely AI2, while 31 blocks were used in the application developed with AI2 with the new *Experience Tracker* and *User* components. By using the specific xAPI components, 326% fewer blocks were required.

**Fig. 14:**
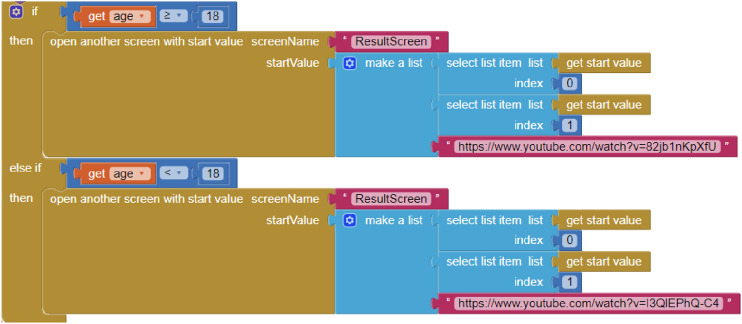
Blocks used to create transitions between different scenarios using AI2 without specific components

Finally, concerning the scene flow management, it is necessary to define as many screens as VR scenarios have been implemented within the application when using only AI2. In addition, as shown in Fig. 14, the app developer must manually create the transitions between the different scenarios through an overload of ”if” conditional control blocks. In this case, the application developed with AI2 required the use of 3 *Screens*, while the application developed with AI2 equipped with the *Workflow* component only needed one single *Screen*. Concerning the blocks used, the application developed with AI2 required 43 blocks to manage the transitions and conditions among different VR scenes, while the application developed with the *Workflow* component required a total of 28 blocks to set up the workflow engine; hence 53% less. With this approach, the scenes and their transitions can change, while the number of blocks required remains unchanged, thanks to the different scenarios and the decision flows are embedded in the BPMN model and not in the program itself.

In summary, to develop the application using the base AI2, 232 blocks were needed, while the same application developed with AI2 and the new components required only 77 blocks to support all the features. This overall difference represents 201% fewer blocks needed using the software contributions presented in this paper. The files corresponding to the two implementations of the app are provided as annexed material (https://figshare.com/s/6f3593f0488f98aea745.).

## A multimodal app for foreign language learning

In order to demonstrate the feasibility of the AI2 extensions described in this paper, this section presents a case study with a mobile app to enable foreign language students to immerse themselves in real-world conversations with native speakers of the target language. The study was carried out in an introductory course of German as a foreign language that took place at the University of Cadiz in 2018. In this course, 24 students participated. Firstly, the requirements and design of the app are explained, then a user satisfaction analysis is presented, and finally, a learning analytics study to examine the student behaviour is described.

### App requirements and design

To prove the suitability of our contribution for creating VR-based experiences, a game-based German language learning app, called *Let’s date!* has been developed in collaboration with the instructors of an introductory German foreign language course at the University of Cádiz. The app recreates the environment of a dating agency, providing its users with the opportunity to practice their oral skills (listening, speaking, and pronunciation). Employing personal interviews, a so-called agency assistant tries to help the users to find their ideal partner. To this end, the app requires its users to interact with the agency assistant via voice commands for answering a series of questions related to different personal data and characteristics (i.e. gender, age, physical appearance, etc.). All questions are delivered in the form of panoramic videos that have previously been recorded with a German language student taking the role of a data agency assistant. Additionally, to cover different user-profiles and needs, when searching for the ideal partner, a variety of videos with different kinds of questions have been recorded so that depending on the responses given by a user, different videos and questions are delivered.

**Fig. 15:**
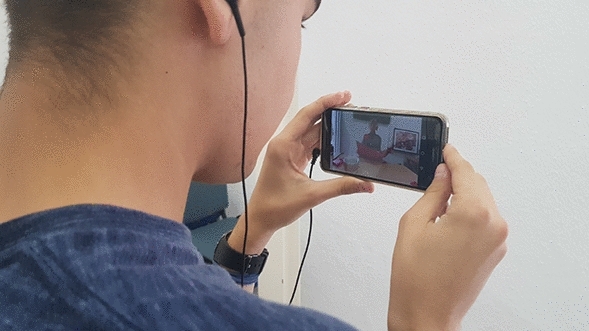
Student using the *Let’s date!* app

In terms of technical requirements, the app can be used either with VR headsets or without them, allowing for semi as well as fully immersive learning experiences (see Fig. 15).

**Fig. 16:**
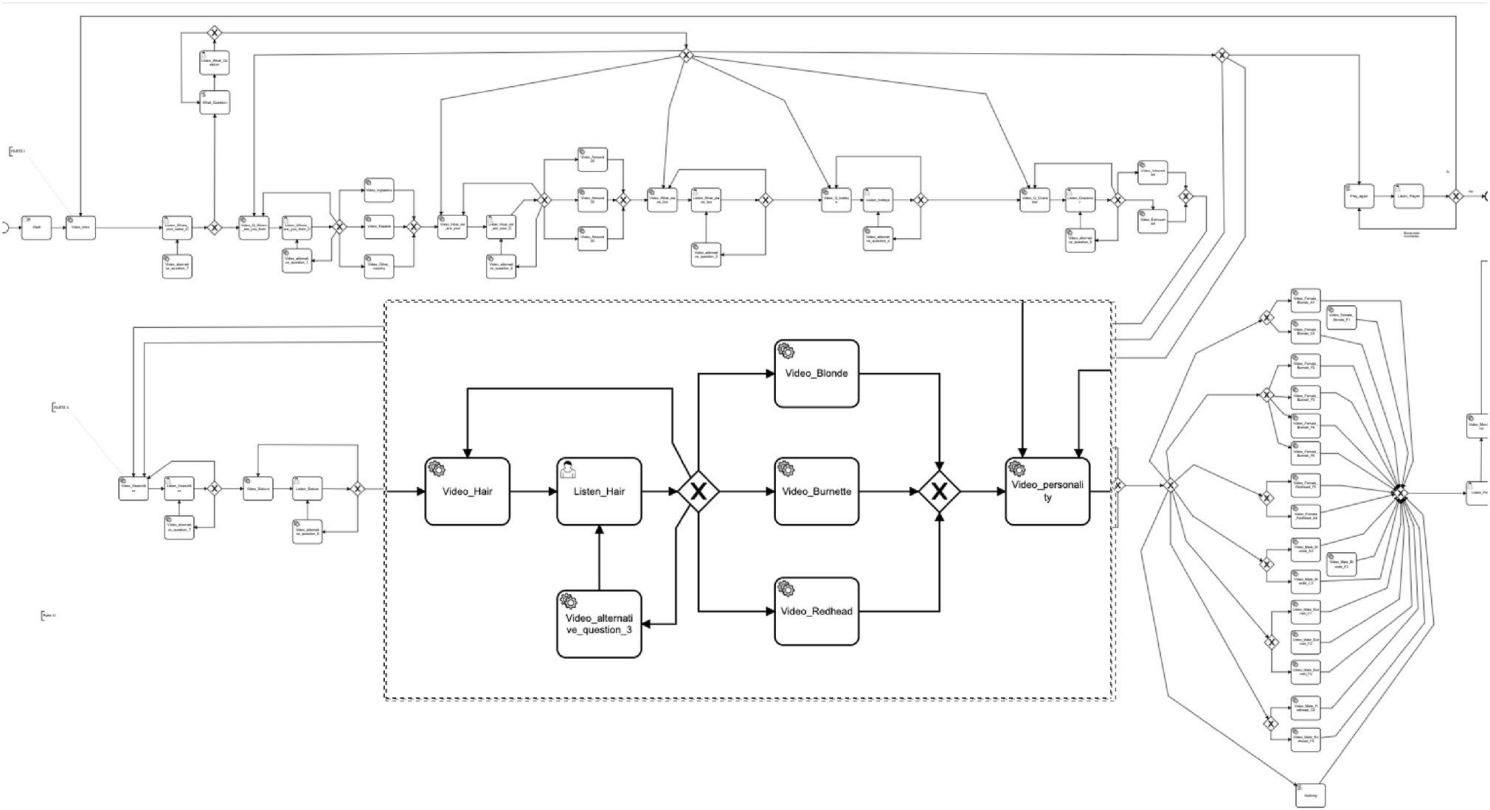
BPMN model for Let’s date! app [https://figshare.com/s/1056588529730282e402]

**Fig. 17:**
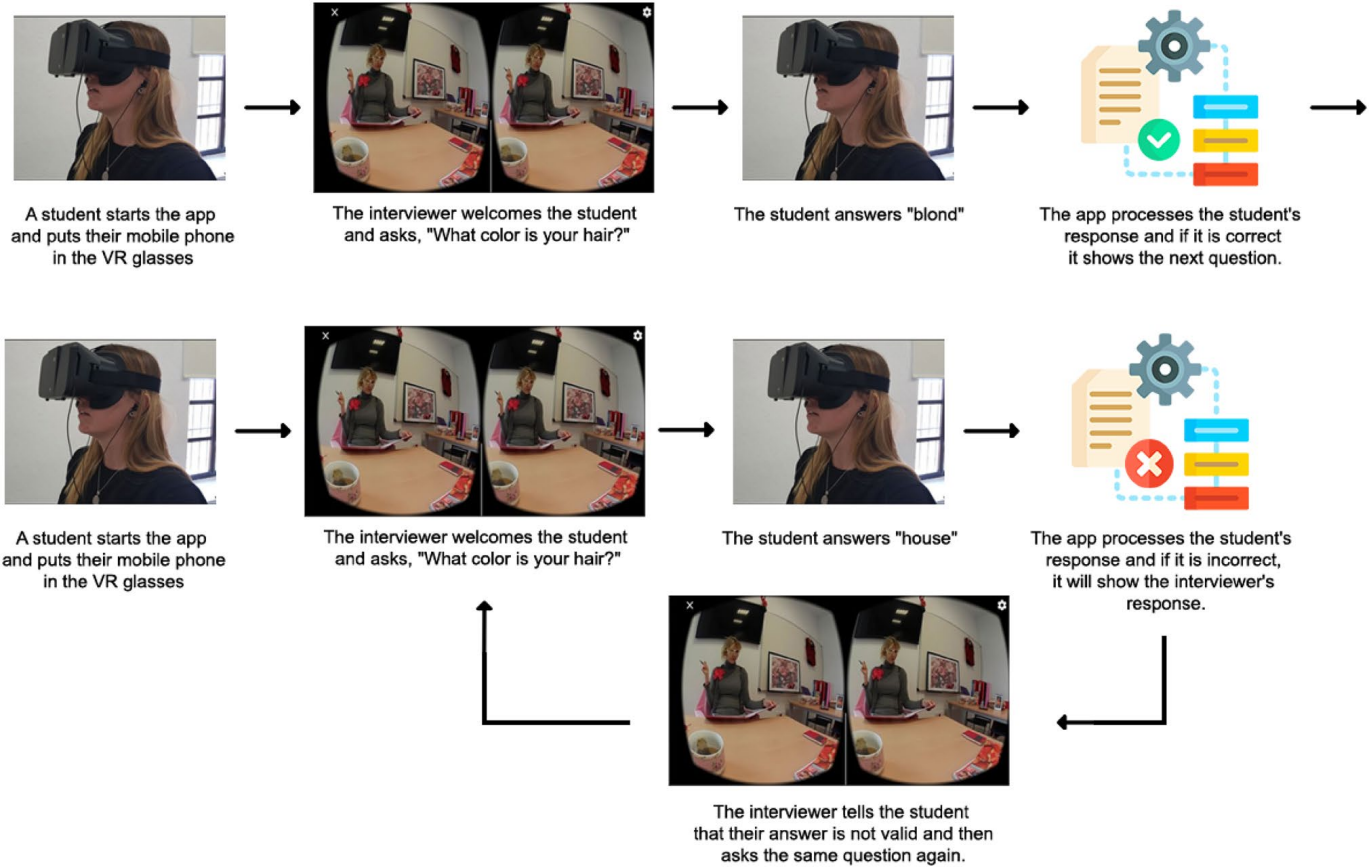
Example of using the Let’s date! app

Regarding its implementation, the developed BPMN process model consists of around 70 tasks for coordinating video delivery (tasks named Video_xyz) and voice comprehension (tasks named Listen_xyz) and 25 gateways for managing flow decisions. With the idea of illustrating the process, Figs. 16 and 17 depict the app model by highlighting a concrete fragment of the BPMN model. The fragment shows the different questions related to the app user’s preferences regarding a specific (i.e. the hair colour and length) aspect and physical characteristic of his/her ideal partner.

The *ExperienceTracker* and *User* blocks are used to send xAPI statements throughout each app run. For this experience, the Learning Locker open-source LRS was used. In these statements the actor parameter includes the student’s mail account and the verb parameter contains the URI of an xAPI verb, as follows:

- On starting the app: http://activitystrea.ms/schema/1.0/start
- On properly abandoning the app: http://activitystrea.ms/schema/1.0/leave
- On successfully reaching the end: http://activitystrea.ms/schema/1.0/win
- On every workflow node change: http://activitystrea.ms/schema/1.0/update
- On every listening action: http://adlnet.gov/expapi/verbs/answered.

The object parameter is supplied with an ad-hoc URI (http://vedils.uca.es/xapi/activities/LetsDate). Furthermore, all the xAPI statements convey additional extensions with app metadata, such as the Java package name of the generated app. Additionally, specific object extensions are supplied, including the workflow current node and the set of voice responses given by the user.

### Student satisfaction analysis

The app was tested with all the students of a 24-member introductory German language course at the University of Cadiz. All learning contents of the app were designed in line with the target students’ language proficiency and curriculum requirements of the first level in the Common European Framework of Reference (CEFR A1).

The students used the app in the classroom, removing the tables so as not to suffer any physical damage during the experience. In addition, each student was provided with virtual reality glasses equipped with a smartphone with the Let’s date app installed. During the experience, their teacher solved doubts about the app’s operation.

Once the experience was over, a Technology Acceptance Model (TAM) [35] survey was provided to the students to obtain information about the app’s acceptance. The TAM model is one of the best-known technology acceptance models originally proposed by Davis et al. [35]. This model suggests that intention to use is determined by two main factors: Perceived Usefulness (PU) and Perceived Ease of Use (PEU). PU refers to the degree to which a person believes that using a given system will improve his or her job performance, while PEU refers to the degree to which a person believes that using a given system will be effortless.

Results were promising since the data obtained from TAM survey revealed that 18 out of 24 students perceived the use of the app as easy and very easy (Perceived ease of use) while only 6 students considered its level of difficulty as normal. However, none of the students perceived the use of the app as difficult [36].

Equally positive was students’ evaluation of the app regarding its usefulness for learning German (Perceived usefulness). Thus, 23 out of 24 students considered that the app helped them boosting their language learning in general, 22 of which stressed especially the app’s potential for improving their comprehension skills, followed by 21 who stressed its potential for improving their pronunciation, 20 their oral expression and 21 their pronunciation. Moreover, apart from boosting different language skills, 20 students considered the app as very useful for their vocabulary learning.

When asking students about their attitude towards using VR apps such as Let’s date! 22 out of 24 students showed a very positive attitude and high interest in using this kind of apps to support their language learning (Behavioural Intention). Among the reasons for their positive attitude, students mentioned especially the app’s motivational potential as well as its interactive and enjoyable nature.

Another aspect that was analysed by the TAM survey focused on the app’s playfulness (Perceived Playfulness) with a special focus on its potential for creating a sense of immersion, which many language experts have stressed as a key factor to boost language acquisition [37, 38]. Interestingly, 19 students confirmed that they lost all sense of time when interacting with the app, followed by 17 students who pointed out that they felt immersed in the virtual environment, while solely 2 students did not and 5 students who neither did nor did not.

When digging deeper into students’ profiles and experience with virtual environments either for learning or gaming, it stands out that students who scored the feeling of immersion the most were -against the authors’ expectations- regular players of immersive video games and hence, very used to immersive environments. The results, therefore, suggest that students’ positive attitude and feeling of immersion were in most cases not due to the effect of novelty concerning the employed learning tool, as it is often reported in the literature [39], but instead due to the way in which the app captures students’ attention and interest.

### Student interaction analysis

The app for emulating a dating agency collects data about students’ interactions, allowing for further analysis. The current section presents the design and results of a study aimed at determining firstly, time metrics regarding the app’s usage, secondly, the number of students who completed the game, and thirdly, the speaking activities that were the most complex ones for the participants, according to the number of attempts made. For that purpose, process mining techniques were used.

#### Data pre-processing

Before analysing the data, a pre-processing stage was conducted to clean and prepare the data. Firstly, the xAPI log stored in the Learning Locker system was exported to a CSV file. This export includes the following data fields: *statement.timestamp*, *statement.actor.mbox*, *statement.verb.id* and *statement.object.definition.extensions*. Then, a data flow was designed by using Pentaho Data Integration (PDI). This process carries out a set of integration tasks (steps), namely:

1. Load the CSV file;
2. Remove URIs of the *statement.verb.id* and *statement.object.definition.extensions* values, maintaining only the final segments;
3. Extract from the *statement.object.definition.extensions* value the current node;
4. Remove events corresponding to the BPMN gateways;
5. Remove events corresponding to the app testing phase (events produced out of the validity time).
6. Generate the *Activity* value by concatenating *statement.verb.id* with the workflow current node;
7. Generate the *Timestamp* value from *statement.timestamp*;
8. Generate the *Resource* value from *statement.actor.mbox*;
9. Generate the *Case* identifier by providing a new numeric identifier for each set of events preceded by a *start* event;
10. Export events in a new CSV file.

After processing the data with PDI, a total of 3441 events (corresponding to 114 cases) were imported in Disco,^3^ a tool for applying process discovery and performance mining techniques from an event log. Before mining the event log, a performance filter was applied for discarding meaningless cases. These cases correspond with non-successful runs of the app mainly due to connectivity losses with the data network and the headsets’ placement. To do that filtering, we removed the events belonging to cases with a duration of fewer than 50 s, mostly containing incomplete sequences of events, such as cases with a single *start* event, or with only the start and *update/Video_intro* events, or with only the *start* and *leave* events, among others. These kinds of events, which are semantically correct but not valid for our analysis, were easily discovered by applying the sequence analysis techniques provided by Disco.

#### Data analysis

The final dataset contains 3376 records belonging to 54 unique case instances. This dataset was analysed with the process mining tool and provided the following results.

Firstly, the cases range from 1 min (the shortest app execution) to 15 min and 10 secs (the longest app execution) with a mean case duration of 6.6 min and a median of 7.2 min.

Secondly, only 31 out of 55 cases were completed. This result was obtained by removing the cases in which the first and last events do not have the *start* event and the *end* event. The successful cases range from 5 min, 56 s to 15 min and 10 s, with a mean case duration of 8.5 min and a median of 8 min.

**Fig. 18:**
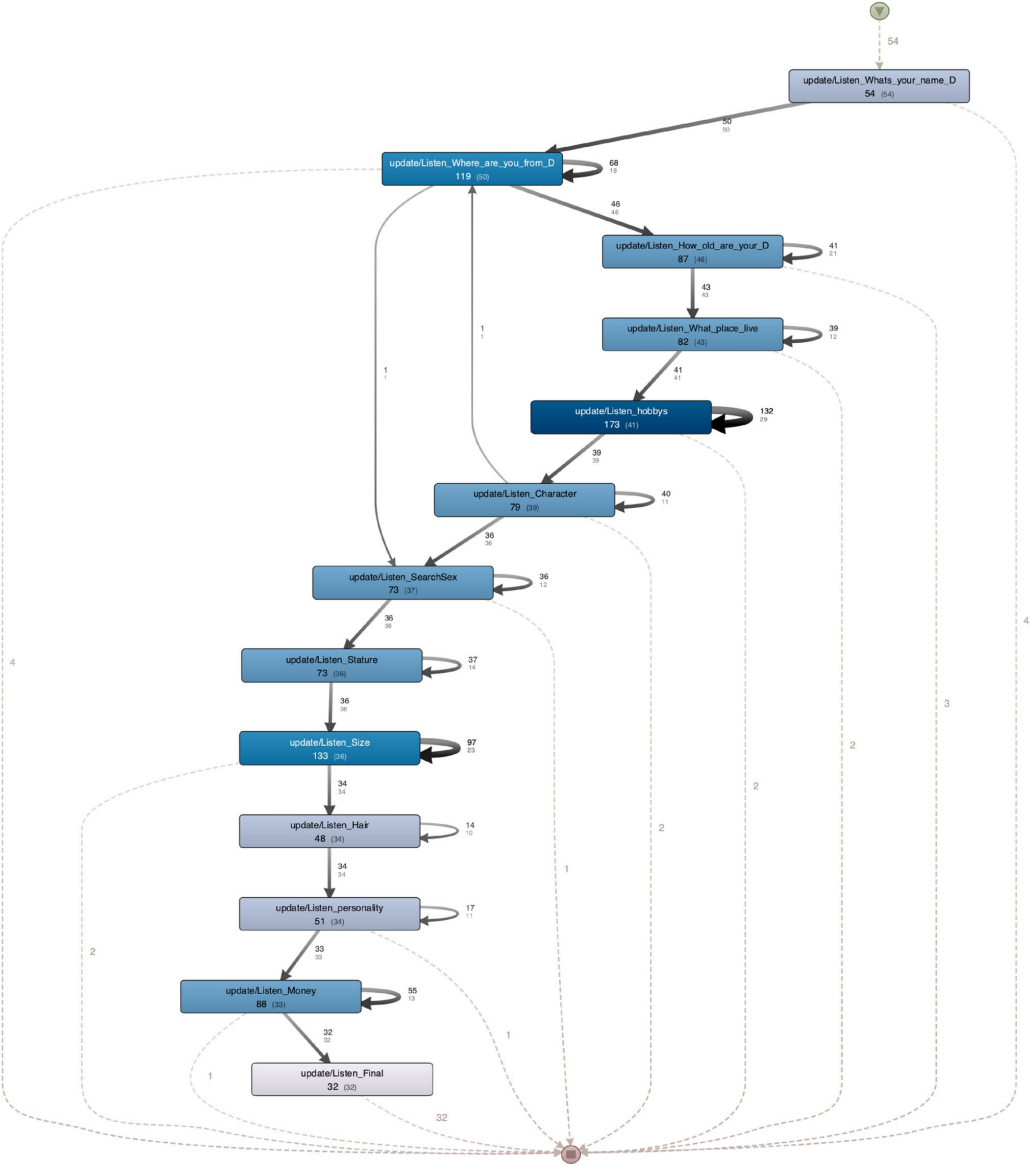
Voice input event model [https://figshare.com/s/1056588529730282e402.]

Finally, intending to identify which were the most difficult questions for the students, we apply a filter to only consider the voice input tasks (Listen_xyz). From the resulting dataset, Disco generated the model shown in Fig. 18. This model is also accompanied by statistics containing the absolute frequency of events (main metric) and cases (metric in brackets) for each activity. The average number of repetitions per case was computed, which provided us with some insights into the complexity of the answers. In this case, the questions related to hobbies and size required 4.22 and 3.69 repetitions on average, whereas the rest of the questions ranges from 2.67 to 1.41, excluding the first and last questions, which admit free answers (see Table 2).

The files corresponding to this study are provided as annexed material (https://figshare.com/s/6f3593f0488f98aea745.).

**Table 2:** Frequency of events and cases for each app scene

Activity	Event frequency	Case frequency	Events per Case
update/Listen_Whats_your_name	54	54	1.00
update/Listen_Where_are_you_from	119	50	2.38
update/Listen_How_old_are_your	87	46	1.89
update/Listen_What_place_live	82	43	1.91
**update/Listen_Hobbys**	**173**	**41**	**4**.**22**
update/Listen_Character	79	39	2.03
update/Listen_SearchSex	73	37	1.97
update/Listen_Stature	73	36	2.03
**update/Listen_Size**	**133**	**36**	**3**.**69**
update/Listen_Hair	48	34	1.41
update/Listen_Personality	51	34	1.50
update/Listen_Money	88	33	2.67
update/Listen_Final	32	32	1.00

The most recurrent activities are shown in bold

## Discussion and conclusions

In recent years, due to technological advances, the use of e-learning has experienced great expansion. These advances have also meant that VLEs are increasingly adopting multimodal interfaces such as VR or Chatbots, generating a large amount of data on their use. Although proposals regarding the creation of such type of VLEs by non-expert users have been found in the literature, none of them enable users to design VLEs that both make use of VR and Chatbots and process the data derived from their use.

This paper addresses this gap by describing a development environment for non-expert users which is capable of generating enriched virtual learning apps. This environment is based on AI2 and consists of a set of components for specific purposes. With the VR component, novice programmers can visually create mobile environments based on 360° video scenes, which is not supported, by default, in AI2. Furthermore, the *Dialog* component enables apps to support voice interaction, saving the app developer from struggling with the necessary blocks to communicate with the HTTP API of the NLP platform, thus reducing the number of blocks used by 430% in the example analysed. Additionally, by using the *Experience Tracker* and *User* components, the app developer can easily decide what data they want to collect from user interactions and send to an xAPI-compliant LRS. In the example analysed, 326% fewer blocks than using the AI2 built-in blocks. Finally, the scene behaviour and navigation are managed in runtime through the *Workflow* component, which can be customized by only editing the BPMN model without altering the program code. In the example analysed, the number of blocks required was reduced by 53%, hence considerably reducing the number of ’if’ control blocks necessary to manage the app action flow according to the user’s responses.

The environment has been successfully implemented during the development of an educational app, called *Let’s date!* that was designed for foreign language learners and which emulates a dating agency. In this app, learners immerse themselves in a realistic scenario in which they must answer a series of questions by means of voice recordings. The app’s aim primary goal is to foster students’ comprehension, speaking as well as pronunciation skills. To gather the users’ feedback on the app, student participants were asked to participate in a TAM survey, whose results revealed that students not only considered *Let’s date!* easy to use, but also appreciated its use for learning purposes. Furthermore, with the idea of identifying various aspects related to the use of the app, a study was carried out using different process mining techniques.

Currently, the *Dialog* component provided by VEDILS does not support the whole BPMN specification. Thus, for example, evolving the model developed for the *Let’s date!* app is not such a simple endeavour because the number of tasks and flow decisions grows as the number of questions does. This drawback can be mitigated by including BPMN subprocess support. As future work, the authors of the current work intend to undertake further studies to analyse and improve the usability of the blocks created in VEDILS for VR and to develop other cases of use for more complex scenarios.

VEDILS enables non-professional developers to create, in the context of a human-centred approach, their own VR experiences combined with voice agents, which goes beyond education, providing a suitable alternative in fields such as job training in environments with high risk or requiring special equipment. Thanks to its block-based VPL and the provided components, creating virtual scenarios is now an easier activity, hence reducing the app development costs.

## Footnotes

1 https://tinyurl.com/4p76uawj.

2 https://developers.google.com/VR.

3 https://fluxicon.com/disco/.
